# Treatment With Remdesivir in Two Pregnant Patients With COVID-19 Pneumonia

**DOI:** 10.7759/cureus.14986

**Published:** 2021-05-12

**Authors:** Vinita Singh, Anisha Choudhary

**Affiliations:** 1 Obstetrics and Gynecology, Tata Main Hospital, Jamshedpur, IND

**Keywords:** coronavirus, pneumonia, pregnancy, intensive care, remdesivir, covid-19

## Abstract

The current coronavirus disease 2019 (COVID-19) pandemic has resulted in an unprecedented global healthcare crisis. Pregnant women belong to a vulnerable group with a higher chance of severe disease and need of intensive care. However, there is limited data on treatment options for severe coronavirus disease in pregnancy. Here, we describe two cases of severe COVID-19 infection in pregnancy with radiological diagnosis of COVID-19 pneumonia, who required intensive care treatment. Both were treated with remdesivir and recovered well without any major side effects, with a favorable maternal and fetal outcome.

## Introduction

The coronavirus disease 2019 (COVID-19) pandemic is one of the biggest healthcare crisis impacting global population. Pregnant women all over the world have been infected by this virus with varying degrees of severity.

Remdesivir inhibits severe acute respiratory syndrome coronavirus 2 (SARS-CoV-2) replication by inhibiting RNA-dependent RNA-polymerase [1]. Few preliminary trials have shown the efficacy of remdesivir in shortening the duration and severity of moderate and severe COVID-19 disease in adults [2,3]. Based on these results, emergency use authorization of remdesivir was granted by the United States Food and Drug Administration and Ministry of Health and Family Welfare, India [4,5]. However, pregnant women have largely been excluded from these clinical trials [6], thus making the management of severe COVID-19 in pregnancy more challenging.

Here, we describe two cases of COVID-19 pneumonia in pregnancy, who were admitted to our hospital, met the criteria for compassionate use protocol of remdesivir, and were successfully managed by an integrated multidisciplinary approach.

## Case presentation

Case 1

**Figure 1:**
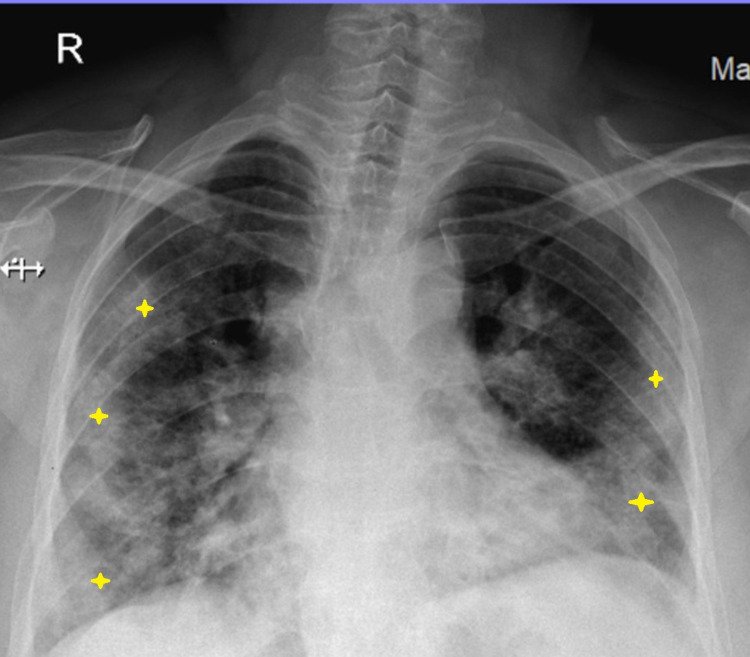
Chest X-ray showing bilateral radio-opacities suspicious for COVID-19 pneumonia. COVID-19: coronavirus disease 2019

A 29-year-old primigravida at 32 weeks of gestation was admitted with chief complaints of fever, cough, and breathlessness for five days. She had no significant past medical history and had regular antenatal visits. On admission, the patient was febrile with a temperature of 101.8°F, blood pressure of 112/74 mmHg, tachypneic to 25 breaths/minute, and with oxygen saturation of 92% on room air. Her chest X-ray showed bilateral radio-opacities suspicious for COVID-19 pneumonia (Figure 1).

She was admitted in the isolation ward and started on supplemental oxygen inhalation. Initial investigations on admission showed neutrophilic leukocytosis, thrombocytopenia, and increased values of C-reactive protein (CRP), serum ferritin, and D-dimer levels (Table 1).

**Table 1:** Laboratory investigations of the patient on days of illness. HD: hospital day; CRP: C-reactive protein; LDH: lactate dehydrogenase; TLC: total leukocyte count; ALT: alanine transaminase; AST: aspartate aminotransferase

Parameter	HD 1	HD 2	HD 4	HD 6	HD 8	HD 10	Normal range
CRP (mg/dL)	22.4	26.5	28	12	4.10	1.8	0.08-0.79
Serum ferritin (ng/mL)	350.8	-	-	270.90	-	-	15-300
LDH (U/L)	392	-	-	307.8	-	-	208-378
TLC (cells/mm^3^)	7,900	-	-	9,500	-	12,000	4,000-11,000
Hemoglobin (g/dL)	11.9	-	-	-	-	10.6	11.5-16.5
Platelet (cells/mm^3^)	96,000	-	-	138,000	-	204,000	150,000-410,000
D-Dimer (ng/mL)	446	-	-	-	-	-	<250
ALT (U/L)	-	38.4	116.2	146.3	119.5	69.2	5-40
AST (U/L)	-	23.8	128.1	146.5	143.1	48.2	5-45

She was started on supportive management and oxygen inhalation was continued, with the goal of keeping oxygen saturation ≥95%. Patient tested positive for COVID-19 (by rapid antigen test), her oxygen requirement increased, and she required non-invasive ventilation support. She was transferred to COVID-19 positive intensive care unit (ICU) for further management. On the second day of admission, a multidisciplinary decision of starting injection remdesivir was taken as her oxygen requirement was increasing, and her inflammatory markers showed an upward trend. As the teratogenic effects of remdesivir are not completely known, the risks and benefits of starting remdesivir was explained to the patient and an informed consent was taken. She was also started on injection dexamethasone and injection enoxaparin in the background of moderate disease with increased D-dimer levels. Her liver enzymes were monitored which peaked on day six of starting remdesivir but were never higher than five times of upper limit of normal range, hence remdesivir was continued. An ultrasound was done to examine the fetus which was within normal limits. On day seven of hospitalization, her oxygen requirement started to decrease, and other inflammatory markers showed a downward trend (Table 1). On day eight of admission, the patient was shifted to the general medical ward and started to maintain saturation in room air from day 10 onward. She was discharged on day 11 after completing 10 days of remdesivir. She was followed up telephonically and reported oxygen saturation levels of >95% on self-monitoring. Patient had a preterm vaginal delivery at 36 weeks of gestation in a district hospital.

Case 2

A 32-year-old gravida 2, para 1 at 29 weeks and three days of gestation presented in our emergency department with fever for four days and altered sensorium and confusion for two to three hours. She had no prior history of any medical disorder. On examination, she was afebrile, pulse rate was 80 beats/minute, blood pressure was 120/82 mmHg, respiratory rate was 14 breaths/minute, and oxygen saturation on room air was 86%. Her random blood sugar was 138 mg/dL. The patient was admitted in the isolation ward and started on oxygen support to maintain saturation of ≥95%. Initial investigations showed leukocytosis with neutrophilia, elevated CRP, lactate dehydrogenase (LDH), serum ferritin, and D-dimer levels (Table 2).

**Table 2:** Laboratory investigations of the patient on days of illness. HD: hospital day; CRP: C-reactive protein; LDH: lactate dehydrogenase; TLC: total leukocyte count; ALT: alanine transaminase; AST: aspartate aminotransferase

Parameter	HD 1	HD 2	HD 4	HD 6	HD 8	HD 10	Normal range
CRP (mg/dL)	18.2	-	11.5	-	-	2.2	0.08-0.79
Serum ferritin (ng/mL)	383	-	-	-	106	-	15-300
LDH (U/L)	382	-	-	-	225	-	208-378
TLC (cells/mm^3^)	13,200	-	8,700	-	5,500	-	4,000-11,000
Hemoglobin (g/dL)	10.8	-	-	-	-	10.6	11.5-16.5
Platelet (cells/mm^3^)	105,000	-	90,000	-	-	108,000	150,000-410,000
D-Dimer (ng/mL)	576	-	-	-	-	-	<250
ALT (U/L)	143	-	121	-	131	115	5-40
AST (U/L)	164.2	-	137	-	151	122	5-45
Sodium (mEq/L)	139	-	141	-	-	140	136-146
Potassium (mEq/L)	3.1	-	3.3	-	-	3.6	3.5-5.5
Blood urea (mg/dL)	19.6	-	19.1	-	-	23.3	115-40
Serum creatinine (mg/dL)	0.54	-	0.51	-	-	0.53	0.5-1.5

**Figure 2:**
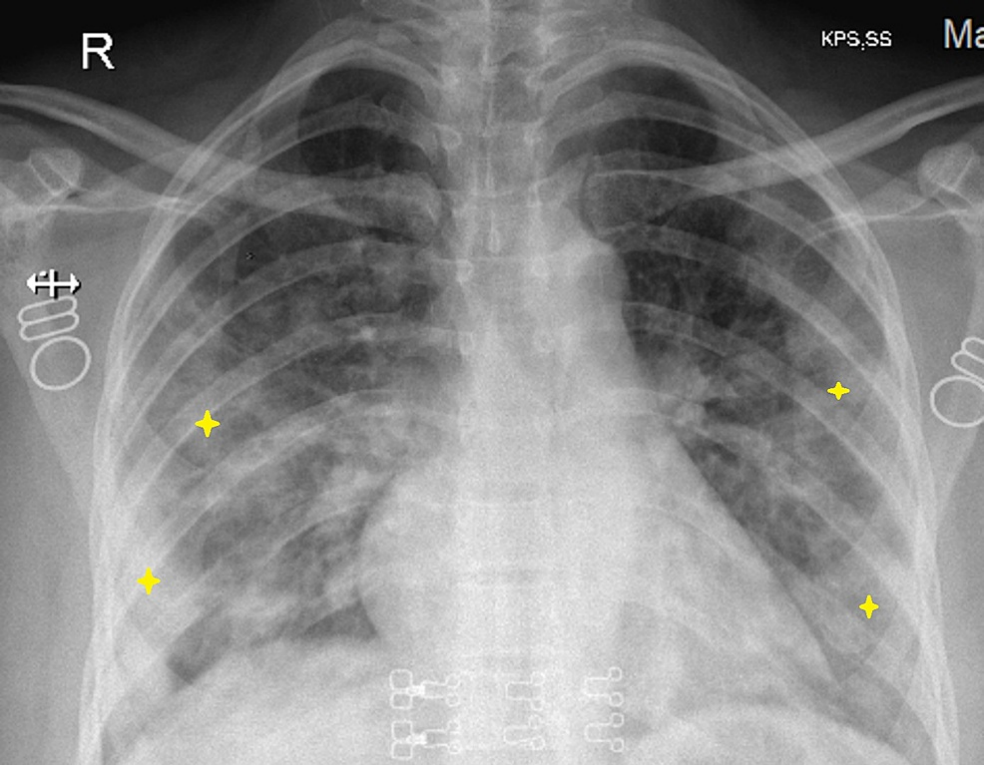
Chest X-ray showing diffuse radio-opacities bilaterally suggestive of COVID-19 pneumonia. COVID-19: coronavirus disease 2019

Her nasopharyngeal swab for rapid antigen test was taken which tested positive for COVID-19. A chest X-ray was done which showed diffuse radio-opacities bilaterally (Figure 2).

After admission, the patient had fluctuating levels of orientation. Motor examination revealed normal tone and deep tendon reflexes. Bilateral plantar reflexes were flexor. There was no neck stiffness or any other evident signs of meningitis. A neurological opinion was taken and a probable diagnosis of COVID-19 encephalitis was made. A combined decision of starting her on injection remdesivir, injection ceftriaxone, injection dexamethasone, and injection enoxaparin was taken. She was also started on injection levetiracetam empirically, with suspicion of seizures being a possible cause for her altered consciousness. The patient responded well, and her oxygen saturation level started to improve. Her level of consciousness and orientation improved dramatically. Her liver function tests were monitored, and her other blood investigations started to improve (Table 2). Her oxygen requirement started to decrease from day eight of admission. An ultrasound for fetal wellbeing was done and was normal. The patient was discharged on day 12 after completing 10 days of remdesivir. She was advised to continue tablet levetiracetam and to visit a neurophysician after magnetic resonance imaging (MRI) of the brain, but the patient did not come for follow-up. She was admitted again at 38 weeks of gestation with labor pains and had caesarean section in view of fetal distress. She had no neurological signs and symptoms at present admission and recovered well postoperatively.

## Discussion

SARS-CoV-2, is a positive-sense RNA virus and the causative agent of COVID-19. Recent data have shown that women diagnosed with COVID-19 in pregnancy have an increased risk of ICU admission, need of mechanical ventilation, and mortality compared to non-pregnant women of the similar age group [7]. Therefore, it is crucial that effective treatment options for severe COVID-19 in pregnancy are explored and reported. However, contrary to this need, pregnant women have largely been left out from clinical trials during COVID-19 pandemic [6].

Replication of SARS-CoV-2 depends on the viral RNA-dependent RNA polymerase (RdRp), which is the likely target of the investigational nucleotide analogue remdesivir [1]. Two major trials, the Adaptive Covid-19 Treatment Trial (ACTT-1) by the National Institutes of Health [3] and the SIMPLE study by Gilead Sciences [2] demonstrated the superiority of remdesivir over placebo in shortening the recovery time in patients with COVID-19 infection. However, both these trials were conducted on non-pregnant adults. Recently, few cases have been reported about the use of remdesivir in pregnant patients. Igbinosa et al. reported three cases of severe COVID-19 in pregnancy who responded well to remdesivir treatment with minimal side effects [8].

Here, we report two cases of severe COVID-19 in pregnancy with varied symptoms at presentation, who were treated with remdesivir with a favorable maternal and fetal outcome. As both our patients were in early third trimester with an active fetus, there was no indication for termination of pregnancy and were managed conservatively in intensive care with a goal to keep the oxygen saturation of the mother above 95% for adequate fetal oxygenation.

Hepatic impairment is a common complication seen in patients with SARS-CoV-2 infection and elevated levels of alanine transaminase (ALT) or aspartate aminotransferase (AST) are the most commonly seen manifestations of liver injury in these patients [9]. Elevated transaminases is also one of the noted side effects of remdesivir, and a value of more than five times the upper limit of normal range is usually considered a contraindication for starting remdesivir [10]. Our first patient also reported raised values of ALT and AST post starting remdesivir therapy; however, the levels remained in the acceptable range and returned to normal after two weeks. Our second patient had elevated liver enzymes and thrombocytopenia on admission and diagnosis of hemolysis, elevated liver enzymes, low platelet count syndrome, or intrahepatic cholestasis of pregnancy were considered. However, as she had no pruritus, her blood pressure values were in the normal range, and her bile acid level was normal, hepatitis and thrombocytopenia associated with COVID-19 infection was considered as the possible explanation. Thrombocytopenia is a common finding associated with COVID-19 and is reported in up to 36.2% of positive non-pregnant adults [11].

COVID-19 is a prothrombotic state [12] often associated with raised D-dimer levels. Pregnancy is already a hypercoagulable state and both our patients had high D-dimer levels, so venous thromboembolism prophylaxis was given. Elevated CRP levels were also seen in these patients, and although raised CRP level can be seen in uncomplicated pregnancy [13], higher value is considered an early marker of severity of COVID-19 [14]. A decline of CRP level was seen post initiation of remdesivir therapy and this was reflected in the clinical improvement of these patients.

SARS-CoV-2 is reported to affect the nervous system and induce polyneuropathy, encephalitis, and acute ischemic strokes [15]. Our patient improved after we started her on remdesivir therapy. An MRI of the brain was advised but patient did not come for follow-up. As she responded well to treatment with no residual signs, her altered level of consciousnesses and disorientation were possibly due to COVID-19 encephalitis.

## Conclusions

Although no broad conclusions can be made based on our case study, we found remdesivir to be safe and possibly effective in the treatment of severe COVID-19 in pregnant women. In both our cases, oxygen requirement decreased, symptoms subsided, levels of CRP and ferritin came down, and sense of wellbeing increased post initiation of remdesivir therapy. No major side effect was seen except rise in liver enzymes which was self-limiting and returned to baseline in two weeks of follow-up. Fetal wellbeing was monitored and no immediate adverse effect was noted. These cases also emphasize the importance of a multidisciplinary approach and prompt treatment in managing such patients. As the data on severe COVID-19 treatment options is limited in pregnant women, especially from the eastern part of India, these case reports may help in being a part of systematic reviews and to formulate evidence-based guidelines. With the pandemic being far from over, there is an urgent need of well-designed randomized controlled trials to study such investigational therapies for treatment of severe COVID-19 in pregnancy.
